# Mutagenesis of the di-leucine motif in the cytoplasmic tail of newcastle disease virus fusion protein modulates the viral fusion ability and pathogenesis

**DOI:** 10.1186/s12985-023-01985-5

**Published:** 2023-02-09

**Authors:** Qingyuan Teng, Lihua Tang, Yahui Huang, Ruihua Yang, Yizhuo He, Guozhong Zhang, Ye Zhao

**Affiliations:** Key Laboratory of Animal Epidemiology of the Ministry of Agriculture, College of Veterinary Medicine, China Agricultural University, No. 2 Yuanmingyuan West Road, Haidian District, Beijing, 100193 China

**Keywords:** Newcastle disease virus, Fusion protein, Cytoplasmic tail, Di-leucine motif, Virus budding

## Abstract

**Background:**

Newcastle disease virus (NDV) is a highly infectious viral disease, which can affect chickens and many other kinds of birds. The main virulence factor of NDV, the fusion (F) protein, is located on the viral envelope and plays a major role in the virus’ ability to penetrate cells and cause host cell fusion during infection. Multiple highly conserved tyrosine and di-leucine (LL) motifs in the cytoplasmic tail (CT) of the virus may contribute to F protein functionality in the viral life cycle.

**Methods:**

To examine the contribution of the LL motif in the biosynthesis, transport, and function of the F protein, we constructed and rescued a NDV mutant strain, rSG10*-F/L537A, with an L537A mutation using a reverse genetic system. Subsequently, we compared the differences in the syncytium formation ability, pathogenicity, and replication levels of wild-type rSG10* and the mutated strain.

**Results:**

Compared with rSG10*, rSG10*-F/L537A had attenuated syncytial formation and pathogenicity, caused by a viral budding defect. Further studies showed that the LL-motif mutation did not affect the replication, transcription, or translation of the virus genome but affected the expression of the F protein at the cell surface.

**Conclusions:**

We concluded that the LL motif in the NDV F CT affected the regulation of F protein expression at the cell surface, thus modulating the viral fusion ability and pathogenic phenotype.

## Introduction

The Newcastle disease virus (NDV) belongs to the genus *Orthoavulavirus* within the family *Paramyxoviridae*. NDV is a non-segmented, single-stranded, negative-sense RNA virus that can infect more than 250 species of birds and causes serious agricultural problems worldwide [1, 2]. The NDV genome contains approximately 15.2 kb, encoding six structural and two non-structural proteins [3]. The fusion (F) and hemagglutinin-neuraminidase (HN) proteins are surface glycoproteins anchored to the viral envelope that facilitate viral entry into the cells [4].

The complete F protein consists of 553 amino acids and is first synthesized as the inactive precursor F0 and then cleaved into the active forms F1 and F2 by host cell proteases [5]. The F1 subunit consists of an *N*-terminal fusion peptide, which is composed of hydrophobic and short-chain amino acids, and is thought to insert into the target membranes to initiate fusion [6]; two heptad repeat sequences, which affect the folding and fusion activity of the F protein [7]; and a *C*-terminal transmembrane domain that anchors the protein to the membrane of the virus or infected cells [8]. In addition to these external structural domains, the F1 subunit contains a short cytoplasmic tail (CT) extending inside the plasma membrane [9].

The NDV F protein CT is 31 amino acids long and is highly conserved in different strains. It has been previously reported that amino acid mutations in the CT resulted in inhibited syncytium formation and the CT played the key role in the interaction between the F proteins and lipid rafts [10, 11]. *C*-terminal deletions and a single tyrosine substitution in the CT resulted in hyperfusogenic phenotypes and increased viral replication and pathogenesis [12]. The function of F protein CTs in NDVs is similar to that in other paramyxoviruses, such as Nipah virus (NiV), human respiratory syncytial virus (HRSV), and measles virus [13–15]. Motifs in the NiV F protein CT can modulate virus particle assembly and egress [16]. The HRSV F protein CT has been shown to play a critical role in F protein cellular localization and the production of infectious virus particles, which was mediated by F protein–lipid raft interactions [17].

Glycoproteins are synthesized in the endoplasmic reticulum and are transported to the plasma membrane through the secretory pathway. The CT are thought to promote the proper transport of glycoproteins to the cell surface by binding transport motifs to cellular factors [18]. The F protein CT of the NDV has typical transport motifs, including a tyrosine motif (YXXφ) at position 524–527 and a di-leucine motif (LL) at position 536–537 [19]. Recently, the YXXφ motif of the NDV has been shown to be related to the transport of F proteins to the cell membrane surface, which affects the viral pathogenicity [20]. The LL motif has been shown to affect internalization of the virus, targeting specific compartments within cells, and resulting in basolateral targeting in polarized epithelial cells [21–23]. The LL motif in the CTs of other viral envelope glycoproteins has been shown to affect viral fusion and infectivity [24, 25].

In the present study, we successfully rescued an rSG10*-F/L537A strain with a single point mutation in the F protein at L537, and compared the differences in the syncytium formation ability, pathogenicity, and replication levels of wild-type rSG10* and the mutated strain. By exploring the relationship between F protein cell surface expression and viral budding, we elucidated potential mechanisms by which the LL motif affected viral pathogenicity and syncytium formation.

## Materials and methods

### Cells and viruses

DF-1 cells (a chicken embryo fibroblast cell line), and BSR T7/5 cells (a baby hamster kidney cell line stably expressing T7 RNA polymerase) were cultured in Dulbecco’s modified Eagle’s medium (DMEM; Gibco, Grand Island, NY, USA) with 10% fetal bovine serum (FBS; Gibco) and were maintained in DMEM with 2% FBS at 37 °C in a 5% CO_2_ incubator (Thermo Forma, Marietta, OH, USA). The recombinant NDV strain rSG10* with artificially introduced Pme I and Sac II restriction sites was generated from rSG10 and kept in our laboratory [20, 26]. The rSG10* was propagated in 9–11 day-old specific-pathogen-free (SPF) embryonated eggs by allantoic cavity inoculation.

### Plasmid construction and virus rescue

To generate LL-motif mutants F/L536A, F/L537A and F/L536AL537A, mutagenesis PCR were conducted using Fast Mutagenesis System (Transgen Biotech, Beijing, China). Primers used were as follows: F/L536A-F: GGCACAACAAAAGACCGCGCTATGGCTTGGA, F/L536A-R: GGTATTATTTCCAAGCCATAGCGCGGTCTTTTGTTG; F/L537A-F: GGCACAACAAAAGACCTTGGCATGGCTTGGA, F/L537A-R: GGTATTATTTCCAAGCCATGCCAAGGTCTTTTGTTG; F/L536AL537A-F: GGCACAACAAAAGACCGCGGCATGGCTTGGA, F/L536AL537A-R: GGTATTATTTCCAAGCCATGCCGCGGTCTTTTGTTG. LL-motif mutant plasmids were individually inserted into the full-length antigenomic cDNA of strain rSG10* in place of the corresponding NDV F open reading frame (ORF) using the restriction endonuclease sites Pme I and Sac II. Virus rescue was performed as previously described [26]. Briefly, the recombinant viruses were recovered by the cotransfection of BSR T7 cells with the full-length cDNA plasmid and the three helper plasmids encoding NP, P and L protein of NDV. At 4 days posttransfection, the cell culture was harvested after three freeze&thaw cycles. The supernatant was then injected into 9-day-old SPF embryonated chicken eggs through the allantoic cavity. After 4 days incubation, the allantoic fluid was harvested and screened with a hemagglutination (HA) assay. The rescued recombinant virus was sequenced after RNA extraction and PCR. The cDNA encoding rSG10* F protein, or the LL motif with various mutations, was cloned into pRK5-Flag (BD Biosciences) to generate the following plasmids: pRK5-Flag-F, pRK5-Flag-F/L536A, pRK5-Flag-F/L537A, and pRK5-Flag-F/L536AL537A.

### Antibodies

Mouse polyclonal antibody against NP, rabbit polyclonal antibody against F protein, and chicken polyclonal antibody against HN protein were prepared in our laboratory. Anti-Flag (#14793, CST, MA, USA), anti-β-actin (#3700, CST, MA, USA), anti-GAPDH (#2118, CST, MA, USA), and Anti-Sodium Potassium ATPase (Na/K ATPase) (#ab76020, Abcam, Cambridge, MA) were purchased from Univ-bio (Shanghai Univbio Co., Shanghai, China). The secondary antibodies against mouse, rabbit, or chicken used for indirect immunofluorescence assays (IFA) and western blotting were purchased from Bioss Biotechnology (Beijing, China).

### Animals and ethics statement

All SPF embryonated eggs and SPF chickens were purchased from Beijing Boehringer Ingelheim Vital Biotechnology Co., Ltd. (Beijing, China). The Beijing Administration Committee of Laboratory Animals approved the animal experimental protocol under the auspices of the Beijing Association for Science and Technology (approval ID SYXK [Jing] 2018-0038) and Ethical Censor Committee at China Agricultural University (CAU approval no. 2021028).

### Cell transfection and infection

BSR-T7/5 cells grown to 70–80% confluence were transfected with the plasmids using Lipofectamine 2000 (Thermo Fisher Scientific, Waltham, MA, USA) and were collected at specific time points for use in further studies.

BSR cells were infected with NDV at an MOI of 3, 0.1, or 0.01. After adsorption for 1 h at 37 °C, the viral solution was removed and then washed three times with phosphate-buffered saline (PBS). The cells were cultured in maintenance medium in a 5% CO_2_ incubator and then were collected at specific post-infection time points. Viral titers in the cells were determined as previously described by assay of the median tissue culture infective dose (TCID_50_) values.

### Growth kinetics

The multiple-cycle growth kinetics in DF-1/ BSR-T7/5 cells was used to determine the growth kinetics of the NDV strains. Cells in triplicate wells of 24-well culture plates were infected with viruses at an MOI of 0.01. Culture supernatants were collected the at specific time points. The viral titers in the collected supernatants were measured by the endpoint dilution method and expressed as TCID_50_ values.

### IFA

The cells were fixed at specific time points, incubated with anti-flag antibody at 4 °C overnight, then incubated with fluorescein isothiocyanate (FITC)-conjugated secondary antibodies (Bioss Biotechnology; diluted 1:200) at 37 °C for 1 h, observed, and photographed using a Nikon Ti2-E fluorescence microscope (Nikon, Tokyo, Japan).

### Western blotting

The total cell protein was extracted from infected or transfected cells with lysate, and the protein samples were separated using 10% SDS-PAGE, and then transferred to a polyvinylidene difluoride (PVDF) membrane (Amersham Biosciences, Freiburg, Germany). The PVDF membrane was blocked with 5% skimmed milk (wt/vol) at room temperature for 3 h and washed with 0.1% Tween 20 in Tris-buffered saline (TBST), and then incubated at 4 °C overnight with primary antibody. After being washed with TBST, the membranes were incubated with horseradish peroxidase-conjugated secondary antibodies (Bioss Biotechnology; 1:10,000 dilutions) at room temperature for 1 h. Observation of protein bands was performed using enhanced chemiluminescence western blotting detection reagent (CWBIO, Beijing, China).

### RT-qPCR

A total RNA isolation kit (Foregene, Chengdu, China) was used to extract the total RNA from animal tissues or monolayer cells, which was then reverse transcribed into cDNA. RT-qPCR analysis used M5 HiPer SYBR Primeix Estate (Mei5 Biotechnology, Beijing, China). The gene expression was standardized to the expression of the housekeeping gene for β-actin (in BSR-T7/5 cells) or GAPDH (in DF-1 cells). Primers used were as follows: SG10-qNP-F: TTACAACTTGGTCGGGGATG, SG10-qNP-R: CGATATAAACGCATGAGCTG; qβ-actin-F: TGCTGTCCCTGTATGCCTCT, qβ-actin-R: TTTGATGTCACGCACGATTT; qGAPDH-F: ATCACAGCCACACAGAAGACG, qGAPDH-R: TGACTTTCCCACAGCCTTA.

### Fusion assessment

BSR-T7/5 cells were used to detect the ability for syncytial formation. The cells were seeded into 12-well plates and transfected with pRK5-Flag-F, pRK5-Flag-F/L536A, pRK5-Flag-F/L537A, pRK5-Flag-F/L536AL537A, or pCMV-HA-HN or infected with rSG10* or rSG10*-F/L537A at an MOI of 0.1. At specific time points, the cells were washed with PBS, fixed in methanol at room temperature for 20 min, and stained with Giemsa. The stained syncytia were visualized using a Nicon eclipse Ti2 microscrope. The syncytium diameter of each image were measured analyzed with image J software. The average syncytium diameters of wild F transfected or infected groups were set at 100%, and the syncytium diameter of LL-motif mutations were expressed as fusion index, which means the relative percentages to the wild F group.

### Pathogenicity in 3-week-old chickens

The pathogenicity of rSG10* and rSG10*-F/L537Awas investigated in 3-week-old chickens. SPF chickens were separated randomly into three groups and were inoculated with 200 µl of PBS or a dose of 10^5^ 50% egg infectious dose (EID_50_) of virus per bird via the oculonasal route. During the 14-day observation period, the birds were observed every day and scored according to clinical symptoms: healthy, 0; sick, 1; wing drop, paralysis, torticollis, or lack of coordination, 2; prostration, 3; dead, 4. At 3, 5, and 7 dpi, three birds from each group were euthanized, and spleen, proventriculus, duodenum, cecum tonsil, lung, brain, and trachea samples were collected for viral load detection via RT-qPCR. The tissues of chickens killed on the 5th day were fixed and used for histopathological analysis.

### Virus budding assay

BSR-T7/5 cells were infected with viruses at an MOI of 0.1. At 36 dpi, culture supernatants were collected and centrifuged at 5000 × g for 15 min, then layered onto a cushion of 20% (wt/vol) sucrose in PBS and ultracentrifuged at 40,000 rpm at 4 °C for 2 h in a SW41 Beckmann centrifuge tube. The resulting pellets were collected from the bottom and dissolved in 100 µl of Sodium Chloride-Tris-EDTA (STE) Buffer. Samples were boiled and analyzed by western blotting as described above. The amount of protein in the cell lysates and viral particles was estimated from the density of the protein bands using Image J software, and the budding index was calculated as follows: the amount of protein in viral particles/the amount of protein in the corresponding lysates, both normalized to the values obtained with rSG10* protein, which were set at 1.

### Cell surface expression of the mutant virus F proteins

A cell membrane and cytoplasmic protein extraction kit (Beyotime Biotechnology) was used to analyze the expression of the F protein at the cell membrane. BSR-T7/5 cells were seeded into 6-well plates and transfected with a plasmid or infected with viruses. At a specific time point, the cells were collected and the cytoplasmic and membrane proteins of the cells were separated using the extraction kit, and then analyzed by western blotting. The anti-β-actin and Anti-Na/K ATPase were used for cytoplasmic and cytomembrane marker, respectively.

To quantify the F protein cell surface expression levels by flow cytometry, BSR-T7/5 cells were seeded into 6-well plates and infected with the viruses at an MOI of 3. The infected cells were digested with 0.25% Trypsin/EDTA mixture (Beyotime, Nantong, China) at 36 h post-infection (hpi) and centrifuged at 4 °C at 2000 × g for 5 min, and then incubated with rabbit anti F antiserum (1:50 dilution) at 37 °C for 1 h. Subsequently, the cells were washed with PBS three times, incubated for 1 h at 4 °C with 1:200 diluted FITC-conjugated goat anti-rabbit immunoglobulin G antibodies, and analyzed with BD FACSCANTO II. The percentage of F-positive cells was analyzed with FlowJo software.

### Coimmunoprecipitation

BSR-T7/5 cells infected with the viruses were harvested at 36 hpi, then lysed in Pierce IP lysis buffer (Thermo), and then centrifuged at 14000 × g for 10 min. The supernatants were precleared by incubation with Protein A agarose beads (Sino Biological, Beijing, China) for 2 h at 4 °C, then the supernatants were incubated with agarose beads coupled with rabbit polyclonal antibody against F protein. The resulting immunoprecipitates and cell lysates were further analyzed via western blotting analysis.

### Statistical analyses

All data were analyzed using GraphPad Prism software version 5.0 (GraphPad Software Inc., San Diego, CA, USA). Statistical differences between different groups were assessed using one-way and two-way analysis of variance (ANOVA) tests. Statistical significance was set at *P ≤ 0.05, **P ≤ 0.01, and ***P ≤ 0.001.

## Results

### LL-motif mutations affect F protein synthesis and influence syncytium formation after transfection

**Fig. 1:**
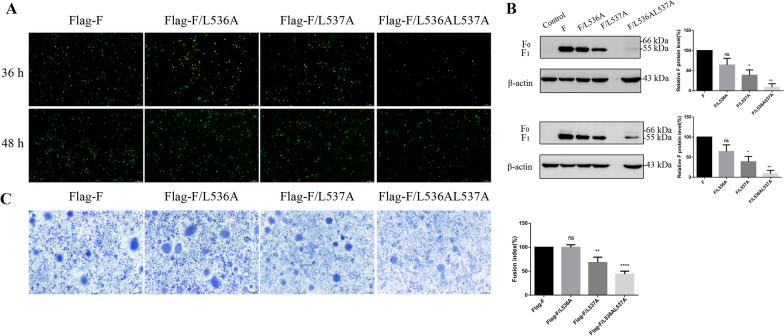
Effect of LL-motif mutations on the F protein synthesis and syncytium formation after transfection. The F protein synthesis of LL-motif mutants was detected by IFA (**A**) or western blotting (**B**). BSR-T7/5 cells were transfected with different LL-motif mutant plasmids and the cell lysates were collected at 36 or 48 hpi. F protein expression levels of LL-motif mutants are expressed as percentages of the levels for F, which were set at 100%. **C** The size of the syncytium induced by LL-motif mutants. After transfection with different LL-motif mutant plasmids, the BSR-T7/5 cells were stained with Giemsa solution to determine the size of the syncytium. The average syncytium diameters of wild F transfected group was set at 100%, and the syncytium diameter of LL-motif mutations were expressed as fusion index, which means the relative percentages to the wild F group. *P* values were calculated with a one-way ANOVA; *n* = 3; *P ≤ 0.05; **P ≤ 0.01; ***P ≤ 0.001

Plasmids expressing the wild-type NDV strain SG10 F protein (WT-F), and mutated F protein with a single amino acid replacement of leucine with alanine at position 536 or 537 (F/L536A or F/L537A), and a variant with a double amino acid replacement (F/L536AL537A), with *N*-terminal Flag tags, were transfected into BSR T7/5 cells. At 36 or 48 h post-transfection, the synthesis of F protein was detected by IFA and quantified by western blotting. As shown in Fig. 1A, B, a significant reduction of F protein expression was found in the F/L537A and F/L536AL537A transfected groups (*p* ≤ 0.05), especially in the F/L536AL537A group in which only a small amount of F protein could be detected at 48 h post-transfection. To investigate whether the decrease in F protein expression affected syncytium formation, we co-transfected the F mutants and a plasmid encoding HN protein into BSR T7/5 cells and found that transfection of F/L537A and F/L536AL537A resulted in a reduction in syncytium diameter at 48 hpt compared with wild type F transfection group (Fig. 1C). In summary, these results demonstrated that LL-motif mutations affected F protein expression and influenced syncytium formation after transfection.

### Construction and rescue of recombinant viruses with LL-motif mutations

To investigate the role of LL motifs in the NDV life cycle, the LL motif-mutated NDV full-length plasmids rSG10*-F/L536A, rSG10*-F/L537A, and rSG10*-F/L536AL537A were constructed. Despite many attempts, only one mutant virus, rSG10*-F/L537A, could be successfully rescued. The mutant virus rSG10*-F/L537A was stably inherited in chicken embryo and expressed NDV-specific protein in BSR-T7/5 cells, while the recombinant virus rSG10*-F/L536A was extremely unstable during passaging and the recombinant virus rSG10*-F/L536AL537A could not be rescued successfully. Therefore, the rSG10*-F/L537A strain was used in all subsequent experiments.

### Mutation of the LL motif attenuated viral pathogenicity

**Fig. 2:**
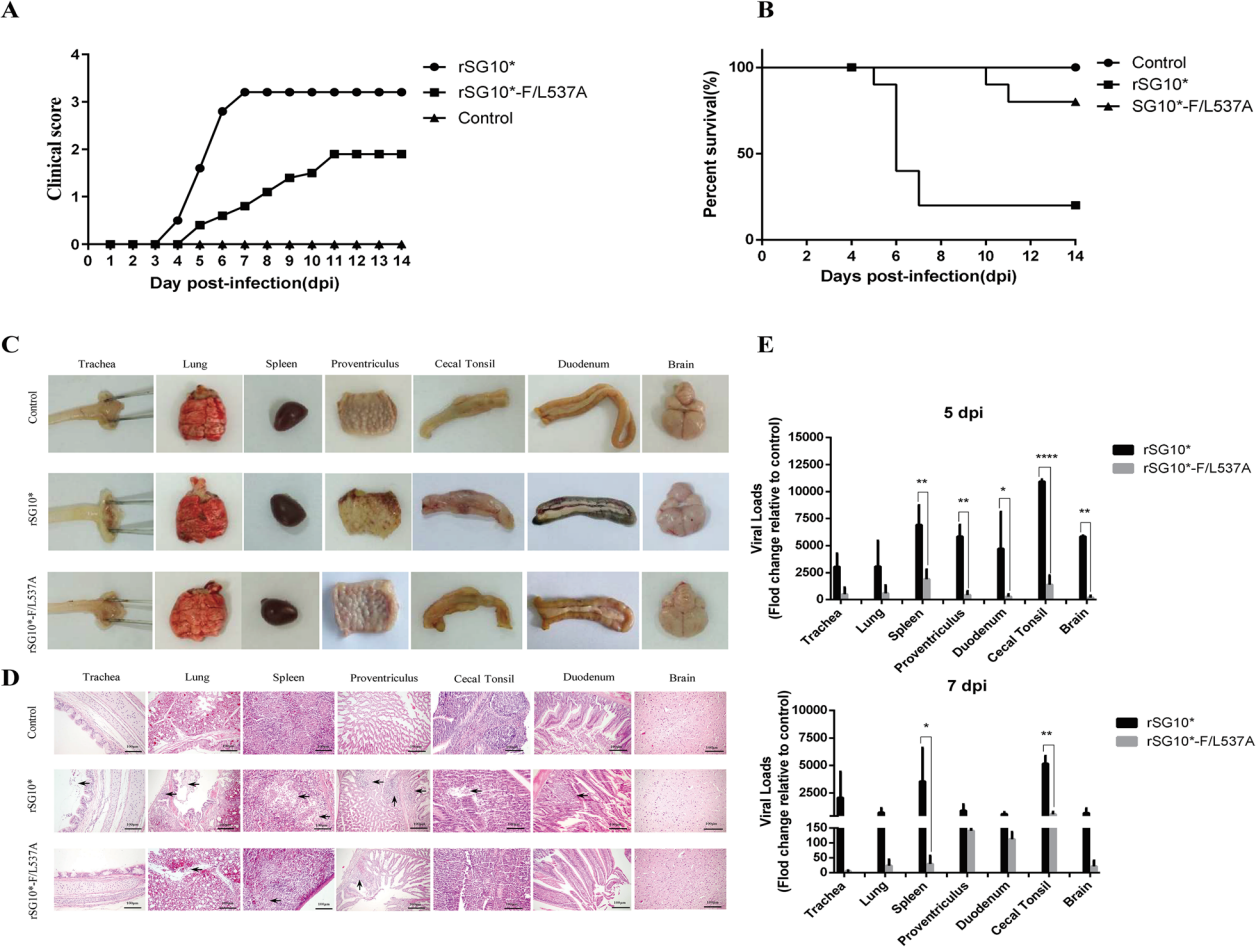
Pathogenicity of rSG10* and rSG10*-F/L537A mutants in 3-week-old SPF chickens. **A** Clinical symptom score of chickens infected with rSG10* or rSG10*-F/L537A (0, healthy; 1, sick; 2, wing drop/paralysis/torticollis/incoordination; 3, prostration; 4, dead); n = 10. **B** Survival curve of 3-week-old SPF chickens inoculated with rSG10* or rSG10*-F/L537A; n = 10. **C** Gross lesions of chickens infected with PBS, rSG10*, or rSG10*-F/L537A at 5 dpi. **D** Histopathology changes in the chickens in each group at 5 dpi. **E** Replication ability of rSG10* and rSG10*-F/L537A in vivo. Three infected chickens in each group were euthanized at 3, 5, and 7 dpi, and indicated tissues were collected to detect the viral loads by RT-qPCR

The pathogenicity of the rSG10*-F/L537A strain in 3-week-old SPF chickens was evaluated. The chickens infected with rSG10* showed typical clinical symptoms at 4 days post inoculation (dpi), which reached a peak at 7 dpi (Fig. 2A). Death of chickens started at 5 dpi and the final mortality rate reached 80% (Fig. 2B). In the rSG10*-F/L537A infection group, only a few of the chickens developed mild clinical symptoms at 5 dpi, prostration was observed in only three of ten chickens during the 14-day observation period, and the final mortality rate was 20%. No clinical symptoms or deaths were observed in the control group (Fig. 2A, B). The pathological damage to target organs caused by rSG10*-F/L537A infections was much slighter than that caused by rSG10* infection. In the rSG10* infection group, gross lesions, including laryngotracheal bleeding, splenomegaly, severe bleeding of glandular gastric papilla, cecal tonsil enlargement, bleeding, duodenal hemorrhage, and necrosis, were observed. In contrast, only some chickens in the rSG10*-F/L537A infection group showed splenomegaly and slight bleeding of the glandular gastric nipple and duodenum (Fig. 2C). Microscopic evaluations of the lesions were consistent with the gross lesions. In the rSG10* infection group, epithelial-cell abscission in the trachea, lymphocyte infiltration in the lungs, massive emptying of lymphocytes in the spleen, necrosis of the lamina propria and glandular tubules in the proventriculus, massive emptying of lymphocytes in the cecal tonsil, necrosis of the lamina propria, and reduction of intestinal glands in the duodenum were observed. In the rSG10*-F/L537A infection group, only a small amount of lymphocyte infiltration in the lung, partial emptying of lymphocytes in the spleen, and necrosis of tissue in the upper lamina propria of the proventriculus were observed (Fig. 2D). The viral load in the target organs of infected chickens was detected by reverse transcription-quantitative PCR (RT-qPCR). As shown in Fig. 2E, the viral loads in the spleen, proventriculus, cecal tonsil, duodenum, and brain in the rSG10*-F/L537A-infected chickens were much lower than those of the rSG10*-infected chickens at 5 dpi. At 7dpi, the viral loads in all the infected groups were decreased, but the viral loads in the spleen and cecal tonsil in the rSG10*-F/L537A group were still lower than those in the rSG10* group. These results indicated that an LL-motif mutation could reduce the pathogenicity of NDV in chickens.

### Mutation of the LL motif affected replication and F protein synthesis of NDV

**Fig. 3:**
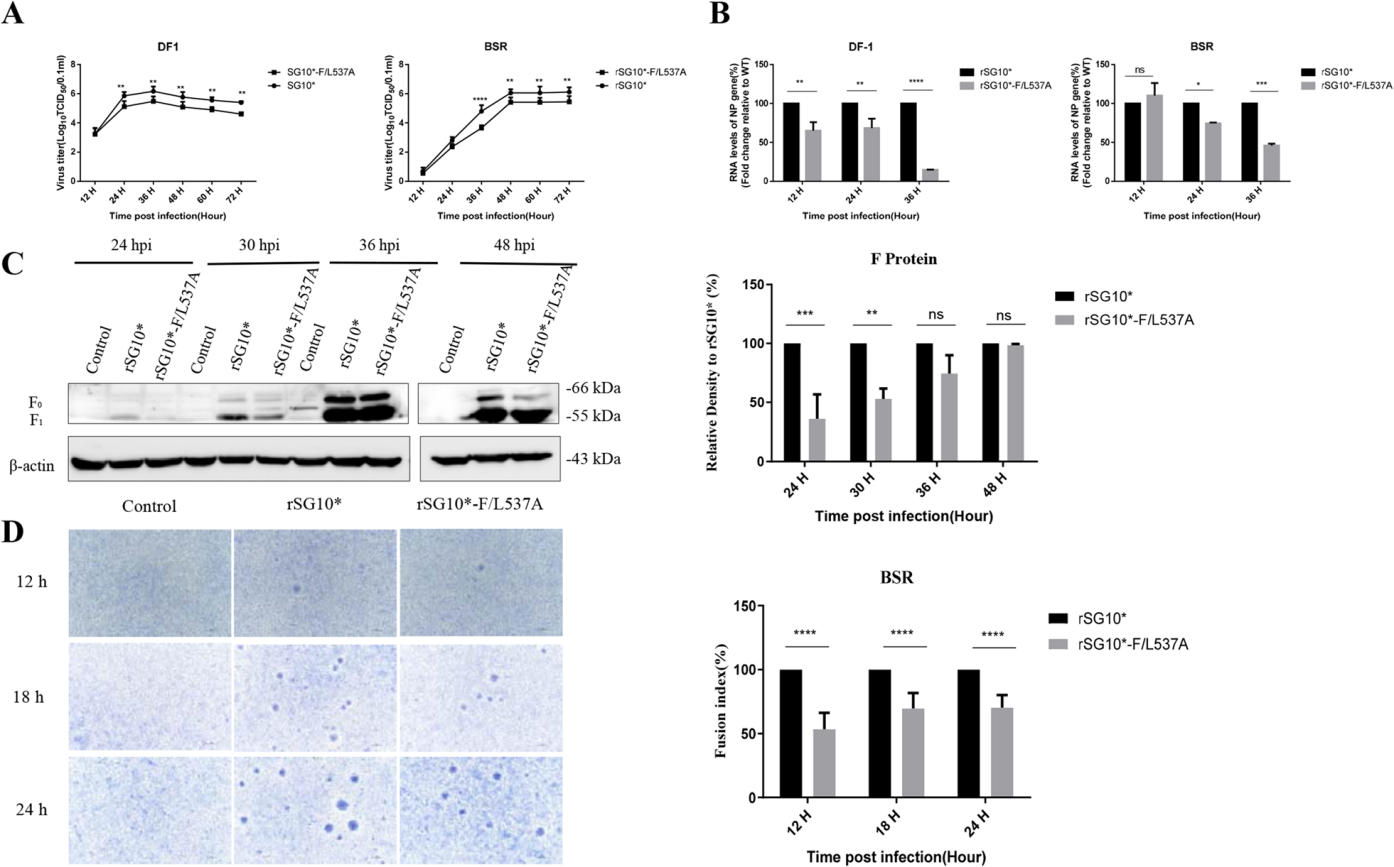
Biological characteristics of the recombinant viruses. **A** The multistep growth kinetics (MOI = 0.01) of rSG10* and rSG10*-F/L537A in DF1 or BSR-T7/5 cells. The supernatants of infected cells were collected at the indicated time point, and the viral loads were quantified as the TCID_50_ values. **B** Replication levels of rSG10* and rSG10*-F/L537A in DF1 or BSR-T7/5 cells. The total RNA levels of the NP gene in monolayer cells were determined by RT-qPCR. RNA levels were normalized to β-actin (in BSR-T7/5 cells) or GAPDH (in DF-1 cells). The total RNA levels of the NP gene are expressed as percentages of the levels for rSG10*, which were set at 100%. **C** The F protein expression levels of rSG10* and rSG10*-F/L537A. BSR-T7/5 cells were infected with virus at an MOI of 0.01 and cell lysates were collected at the indicated times. The F protein expression was analyzed by western blotting using anti-F antibodies. F protein expression levels are expressed as percentages of the levels for rSG10*, which were set at 100%. **D** The sizes of syncytium induced by BSR-T7/5 cells infected with rSG10* or rSG10*-F/L537A. After infection with the viruses, the cells were stained with Giemsa solution to determine the size of the syncytium. The syncytium diameter are expressed as percentages of the levels for rSG10*, which were set as 100% and the ratio was expressed as the fusion index. The average syncytium diameters of rSG10* infected group was set at 100%, and the syncytium diameter of rSG10*-F/L537A infected group were expressed as fusion index, which means the relative percentages to the rSG10* group. *P* values were calculated with a two-way ANOVA; n = 3; *P ≤ 0.05; **P ≤ 0.01; ***P ≤ 0.001

The multistep growth curves of the rSG10*-F/L537A strain were determined by infecting DF-1 and BSR T7/5 cells. The results showed that rSG10*-F/L537A had growth defects compared with rSG10* at 36–72 hpi (Fig. 3A). Next, the total nucleocapsid protein (NP) RNA level of rSG10*-F/L537A was investigated with a multiplicity of infection (MOI) of 0.01. The results showed that the total NP RNA level of rSG10*-F/L537A was significantly lower than that of rSG10* in DF1 cells (after 12 hpi) and BSR T7/5 cells (after 24 hpi) (Fig. 3B). Cells were collected at different time points after infection, and the expression level of F protein was detected by western blotting. The results showed that the expression of F protein in rSG10*-F/L537A was lower than that in rSG10* at the early stage of infection (Fig. 3C). To further investigate whether the decrease in F protein expression caused by LL-motif mutation affected syncytium formation, we determined the size of the syncytium in BSR T7/5 cells infected with recombinant virus at different time points. The results showed that syncytial formation after infection with rSG10*-F/L537A was impaired compared with rSG10* (Fig. 3D). Together these results indicated that the rSG10*-F/L537A mutant inhibited viral replication and protein expression after a low-dose infection, and further affected the formation of syncytium.

### Mutation of the LL motif compromised the viral budding

**Fig. 4:**
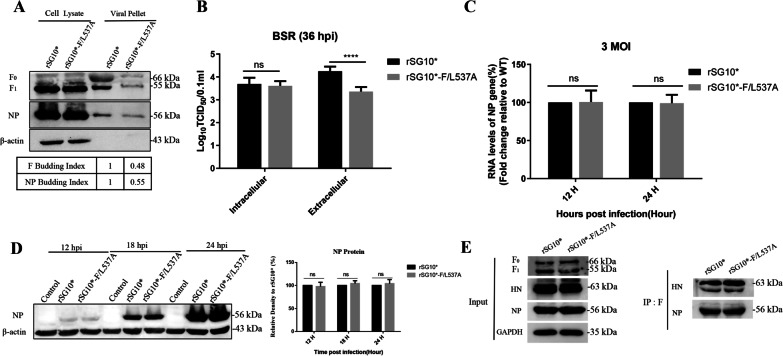
The viral budding, replication, protein synthesis, and assembly of LL-motif mutants. **A** LL-motif mutants caused weakened virus budding. BSR-T7/5 cells were infected with rSG10* or rSG10*-F/L537A. At 36 hpi, the cell supernatants were harvested for ultracentrifugation and the cell lysates and virus particles were analyzed by western blotting. The band intensities were used to calculate budding indices, and all were normalized to rSG10*. **B** Intracellular and extracellular virus titers of LL-motif mutants. BSR-T7/5 cells were infected with rSG10* or rSG10*-F/L537A at a MOI of 0.1, and cell precipitates and supernatants were collected for TCID_50_ analysis. Viral replication (**C**) and translation (**D**) of LL-motif mutants. BSR-T7/5 cells were infected with rSG10* or rSG10*-F/L537A at an MOI of 3, the total RNA levels of NP fragments were determined by RT-qPCR and the NP expression levels were analyzed by western blotting. The total RNA levels of the NP gene and NP protein expression levels are expressed as percentages of the levels for rSG10*, which were set at 100%. **E** Interaction between F and NP or HN protein of LL-motif mutants. BSR-T7/5 cells were infected with rSG10* and rSG10*-F/L537A at an MOI of 0.1, the cell lysates were immunoprecipitated with anti-F antibodies, and then the levels of NP and HN protein were detected by western blotting. *P* values were calculated with a one way or two-way ANOVA; n = 3; *, *P* ≤ 0.05; **P ≤ 0.01; ***P ≤ 0.001

Previous research has shown that the CT region of the F protein plays an important role in the viral budding of paramyxoviruses. Therefore, to explore whether the attenuated pathogenicity of rSG10*-F/L537A was because of the reduced budding of rSG10*-F/L537A, the viral budding efficiency was investigated. After cells were infected with viruses at a MOI of 0.1, the viral protein levels in the cells and the levels of protein incorporated into viral particles in the cell supernatants were determined by ultracentrifugation and western blotting. The results showed that the F and NP protein levels of rSG10* and rSG10*-F/L537A were similar in the whole cell lysates, while in the purified virus supernatants, the levels of the F and NP proteins in rSG10*-F/L537A were lower than those in rSG10* with almost a 50% decrease observed (Fig. 4A). To further demonstrate the effect of the F/L537A mutation on viral budding, the extracellular and intracellular viral titers were quantified for cells infected with viruses at an MOI of 0.1 by determining the medium tissue culture infective dose (TCID_50_). The results showed that there was no significant difference in the intracellular virus titer between rSG10* and rSG10*-F/L537A; however, the extracellular virus titer of rSG10*-F/L537A was lower than that of rSG10* (Fig. 4B). Overall, these results suggested that mutation of the LL motif affected viral budding, resulting in a phenotype with impaired budding.

### The budding deficiency was not related to the ability for viral replication, protein synthesis, or assembly

To further investigate the mechanism of the viral budding deficiency caused by the LL-motif mutation, we examined the effects of the LL-motif mutation on viral replication, protein expression, and viral particle assembly. To eliminate the influence of viral spreading after budding, we used an MOI of 3 for infection to observe a single infection cycle of the virus [27]. We then examined the total RNA and protein expression levels of the viral NP gene at specific time points of infection. The results demonstrated that the viral gene replication and protein synthesis capabilities of rSG10* and rSG10*-F/L537A were similar after infection with high doses of the virus (Fig. 4C). We also examined the effect of the F/L537A mutation on the viral assembly process. After virus infection, the cell lysates were immunoprecipitated with a polyclonal antibody against the F protein, and then the levels of NP and HN proteins were detected by western blotting. The results showed that there was no significant difference in the assembly between rSG10* and rSG10*-F/L537A (Fig. 4D, E). These data suggested that the LL motif mutation-related budding deficiency was not caused by an impairment of the viral replication, protein synthesis, or assembly.

### The expression of F protein on the cell surface of the LL-motif mutant was decreased

**Fig. 5:**
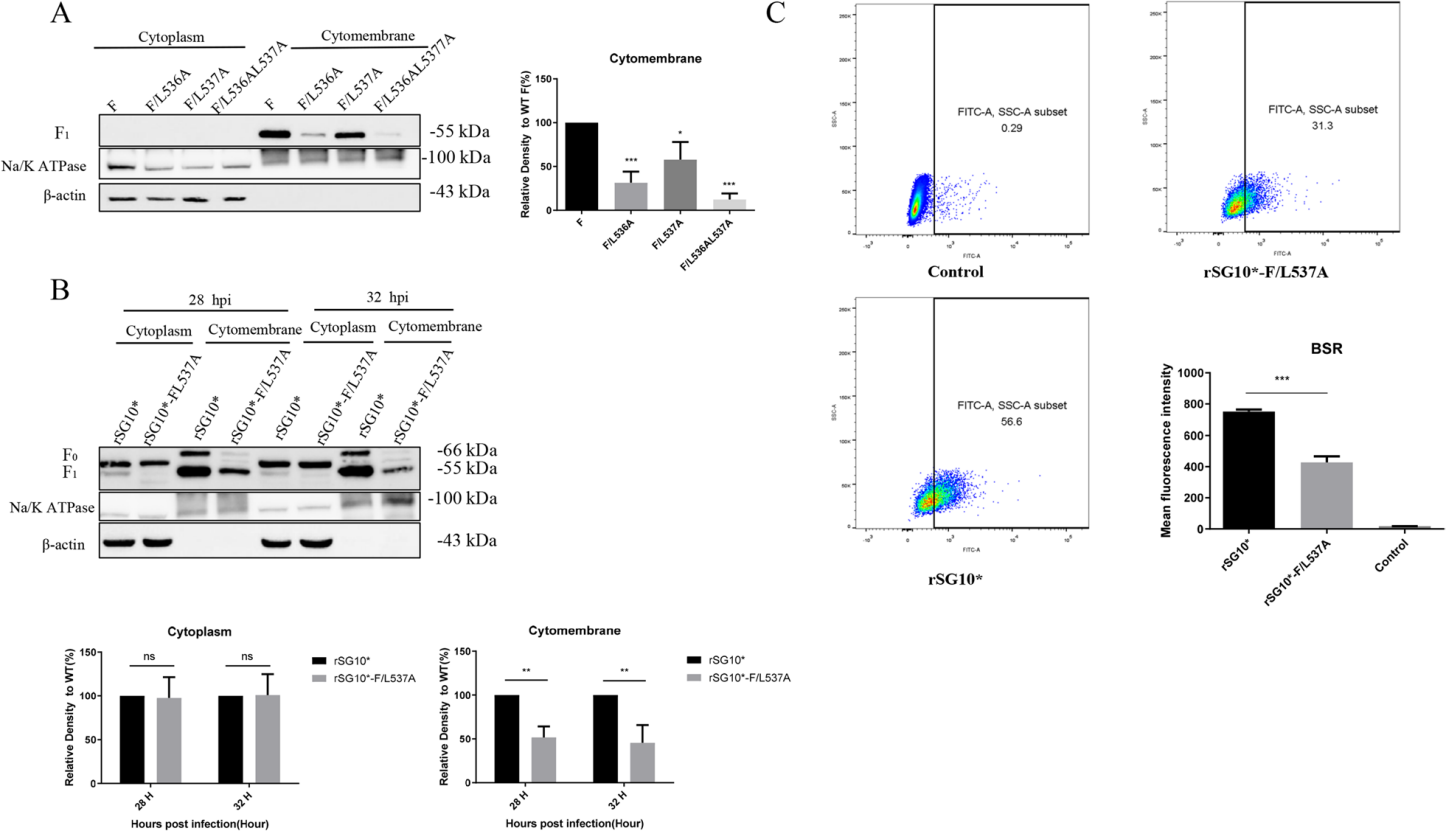
LL-motif mutations affected the expression of F protein at the cell surface. **A** The expression of transfeced F protein at the cell membrane. BSR-T7/5 cells were transfected with different LL-motif mutant plasmids, and the protein was extracted and the level determined at 24 hpi. F protein expression levels of LL-motif mutants are expressed as percentages of the levels for F, which were set at 100%. **B** and **C** Cell surface expression of the F protein of LL-motif mutants. BSR-T7/5 cells were infected with rSG10* or rSG10*-F/L537A at an MOI of 0.1. The cell surface expression of F protein was determined by western blotting (**B**) and flow cytometry assay (**C**) at 36 hpi. F protein expression levels of LL-motif mutants are expressed as percentages of the levels for rSG10*, which were set at 100%. *P* values were calculated with a one way or two-way ANOVA; n = 3; *P ≤ 0.05; **P ≤ 0.01; ***P ≤ 0.001

The F protein is synthesized in the endoplasmic reticulum and processed in the Golgi matrix before being transported to the plasma membrane. Therefore, we investigated whether LL-motif mutations affected the expression of F proteins on the cell surface. We first conducted in vitro transfection as described above. After transfection with LL-motif mutant plasmids, the expression level of F protein on the cell surface was detected by western blotting. As shown in Fig. 5A, the expression of F protein induced by the F/L536AL537A mutant at the cell surface was significantly decreased (*p* ≤ 0.001), while the expression levels with the F/L537A and F/L536A mutants were also decreased to some extent. Next, the F protein expression on the cell surface was also determined after viral infection. After infection, rSG10*-F/L537A induced less F protein expression at the cell surface at 28 and 32 hpi, compared with rSG10*, but the intracellular expression levels were similar (Fig. 5B). In addition, we investigated the expression of F protein on the cell membrane after virus infection by flow cytometry, and the mean fluorescence intensity (MFI) was calculated. The results showed that after the cells were infected with rSG10*-F/L537A, the number of F protein-positive cells and the MFI value were much lower than the values for rSG10*, which was consistent with the trend shown by western blotting (Fig. 5C). In conclusion, these results suggested that the LL-motif mutants resulted in impaired F protein expression on the cell membrane compared with the wild type.

## Discussion

Previous studies have demonstrated that the envelope protein CTs of paramyxoviruses play an important role in viral particle formation, protein folding and oligomerization, and membrane fusion as well as pathogenicity [28–30]. The CTs of envelope proteins contain one or more critical residues related to intracellular transport, viral assembly, and budding [31, 32]. For NDV, according to sequence analysis of the F protein CTs, there are two typical transport motifs: a YXXφ motif and an LL motif, of which the YXXφ motif has been shown to affect the replication and pathogenicity of the virus [20]. In the present study, we evaluated the effect of LL-motif mutations on NDV syncytial formation, infectivity, pathogenicity, and protein transport. We attempted to rescue two single-leucine mutant strains and one di-leucine mutant strain; however, after multiple rounds of plaque purification, we were unable to obtain a homozygous mutant of rSG10*-F/L536A. We were also unable to produce rSG10*-F/L536AL573A despite many attempts, and we speculated that the di-leucine mutation is lethal for the NDV. Subsequently, a pathogenicity assay was conducted in 3-week-old SPF chickens. The pathogenicity of rSG10*-F/L537A was observed to be weakened compared with rSG10*, which was consistent with the corresponding in vitro results.

The NDV mediates the cell-to-cell fusion that is required for the synergistic effect of envelope proteins. The HN protein recognizes sialic acid receptors, and F proteins undergo conformational changes, to jointly drive cell membrane fusion and viral entry [33]. In the present study, the rSG10*-F/L537A mutant showed a hypofusogenic phenotype compared with the wild type. This difference may be because the incorporation of the rSG10*-F/L537A F protein into the purified viral particles was moderately reduced, compared with the wild type. However, other mechanisms may be worthy of further study, such as conformational changes of the F protein or changes in the interactions with the HN protein [34]. For example, in SER virus the enhancement of fusion caused by an N529K mutation in the CT region was significantly related to activation of the fusion protein conformation [35].

The functions of paramyxovirus glycoproteins during assembly and budding are usually related to the motifs in the CTs [36, 37], e.g., the virus-like particle(VLP) formation ability of NiV F CT di-tyrosine trafficking-motif mutants was reduced, compared with the wild type, resulting in a budding deletion phenotype [16]. Therefore, we investigated the effect of the LL-motif mutation in the CT on virus budding to explain the weakened infectivity and pathogenicity of rSG10*-F/L537A. We observed that the extracellular viral titers and the amounts of F and NP proteins incorporated in the virus particles of rSG10*-F/L537A were lower than those for rSG10*. Next, we attempted to elucidate the mechanism by which the LL motif affected viral budding. Because the growth peak of rSG10*-F/L537A was delayed after a low-dose infection, we speculated whether the LL motif affected the replication, transcription, and translation of the virus genome.

According to Poisson distribution, at an MOI of 0.01, the virions could only infect some of the cells as the first batch of viruses need to complete a replication cycle and assemble into complete progeny viruses before starting the next round of infection. Whereas at an MOI of 3, there are enough virions to infect each cell [26]. Therefore, we infected cells at an MOI of 3 to determine the total RNA and protein expression levels of the NP gene. The results showed that the ability for virus replication, transcription, and translation of the rSG10*-F/L537A mutant was not significantly different from that of rSG10*, which indicated that these factors were not related to the budding defect observed with rSG10*-F/L537A.

For several paramyxoviruses, the fusion proteins are transported to the cell surface after synthesis in the endoplasmic reticulum, and this process may be regulated by CTs [38]. Our results showed that the expression of F protein on the membrane surface of rSG10*-F/L537A was significantly lower than that of rSG10*. Therefore, the LL motif may regulate the transport of the F protein from the Golgi to the cell membrane through some mechanism. The short amino acid sequences in the cytoplasmic domain of transmembrane proteins can be recognized by specialized adaptor proteins (AP). The HIV envelope glycoprotein has a highly conserved LL motif in the CT that has been shown to mediate the interaction with an AP and regulate the expression of the glycoprotein on the membrane surface [39]. For NDV, the LL motif may also mediate the binding of the F protein with a specific host protein and regulate the transport of the F protein, but this potential mechanism needs to be further investigated.

The NDV F protein concentrates where lipid rafts gather to participate in the subsequent assembly and budding of the virus [19]. We observed that in the rSG10*-F/L537A mutant, the interactions between the F protein and another envelope protein HN and the NP was not significantly weakened; therefore, the LL-motif mutation may not affect the binding of the F protein to other viral components. From these data, we believe that the effect of the LL-motif mutation on viral budding is mainly caused by a reduction in the amount of F protein transported to the cell membrane; however, there may also be other factors involved that we have not considered.

In conclusion, we have explored the role of the conserved LL motif of the F protein CT in the NDV life cycle. The results showed that an LL-motif mutation could affect the expression of the F protein on the cell membrane, consequently leading to a decrease in the level of budding, resulting in replication defects and attenuated pathogenicity. Our work not only increases the understanding of the function of the LL motif, but also provides a basis for the development of attenuated viral strains. However, the mutation stability and virulence restoration after serial passaging in vivo needs to be further investigated. Also, studies regarding the role of cytokines in the interaction of LL motif of the F protein would be worthwhile.

## Data Availability

All data generated or analyzed during this study are included in the published article.

## Abbreviations

NDV: Newcastle disease virus
NiV: Nipah virus
HRSV: Human respiratory syncytial virus
F protein: Fusion protein
HN protein: Hemagglutinin-neuraminidase protein
NP: Nucleocapsid protein
LL: Di-leucine
CT: Cytoplasmic tail
VLP: Virus-like particle
MFI: Mean fluorescence intensity
AP: Adaptor proteins
SPF: Specific-pathogen-free
dpi: Days post inoculation
MOI: Multiplicity of infection
TCID_50_: The medium tissue culture infective dose
EID_50_: 50% Egg infectious dose
FBS: Fetal bovine serum
DMEM: Dulbecco’s modified Eagle’s medium
STE buffer: Sodium Chloride-Tris-EDTA Buffer
ORF: Open reading frame
PBS: Phosphate-buffered saline
FITC: Fluorescein isothiocyanate
PVDF: Polyvinylidene difluoride
TBST: Tris-buffered saline
